# The Rise of Digital Twins in Healthcare: A Mapping of the Research Landscape

**DOI:** 10.7759/cureus.65358

**Published:** 2024-07-25

**Authors:** Sneha M Kuriakose, Jeena Joseph, Rajimol A, Reji Kollinal

**Affiliations:** 1 Department of Computer Applications, St. Peter’s College, Kerala, IND; 2 Department of Computer Applications, Marian College Kuttikkanam Autonomous, Kuttikkanam, IND; 3 Department of Computer Applications, Baselios Poulose II Catholicose (BPC) College, Piravom, IND

**Keywords:** vosviewer, biblioshiny, bibliometric analysis, healthcare, digital twin

## Abstract

The place of digital twin technology in any healthcare system is a truly disruptive innovation that has profound consequences all across medical research and practice. Digital twins represent the virtual replicas that correspond to some physical entity by pulling real-time data streams from different sources to model biological systems for health monitoring and personalization of treatment strategies. This paper presents a detailed review of the current research landscape into digital twins for healthcare. Through bibliometric analysis, we obtained 1,663 publications from 2012 to 2024, basically sourced from the Scopus database, establishing a portion of the trends, productive authors, influential sources, and collaboration networks in this fast-evolving field. Descriptively, our results indicate that although research into this area started way back, the bulk of research began to be realized from 2018 onwards, with appreciable contributions coming in from interdisciplinary fields of artificial intelligence, machine learning, and data analytics. Even with challenges to data interoperability and other privacy concerns, this change brought on by digital twin technology is undoubtedly a considerable promise for chronic disease management, predictive analytics, drug discovery, and surgical planning. This work brings immense insight into this new domain of digital twins in health, which shall set up a strong foundation for future research and innovation in this area.

## Introduction and background

Over the past years, the concept of digital twins has gained ground in healthcare with a compelling vision of transforming medical practice, research, and patient care. Initially developed in manufacturing to make digital twins of physical assets to optimize their performance and anticipate scheduled maintenance, it is now applied in healthcare by modeling biological systems and monitoring patients' health in real-time to personalize treatment strategies.

The digital twin in healthcare can be defined as the acquisition of data from several sources, such as wearable devices, electronic health records, imaging modalities, and genomic profiles, onto a model that virtually represents the patient or any other state biological system [1]. This is realized on a virtual model that independently tracks and simulates physiological processes. It facilitates the anticipation of health issues in advance, optimization of treatment plans, and change of individual interventions by health providers [2]. Current advances in data science, AI, and sensor technologies can be harnessed for developing digital twins that model complicated interactions within the human organism at an accuracy and granularity previously unattainable [3]. The opportunities this affords for health management range from the management of chronic diseases to predictive analytics, drug discovery, and surgical planning [4].

They have been shown to improve diagnostic accuracy through resultant real-time tracking and predictive modeling of disease progression [5,6]. For instance, the digital twin has had applications in cardiac dynamics simulation for managing cardiovascular conditions at a personalized level [7,8]. Furthermore, sophisticated data analytics has been realized by integration with AI algorithms that spot, early enough, anomalies and inform proactive interventions [9]. Notwithstanding these achievements, interoperability issues across heterogeneous sources and those concerning data privacy and security remain [10,11]. On the other hand, further research is constantly pushing the limits of what is doable in using digital twins for healthcare toward optimizing treatment outcomes and thus delivering patient-centered care.

Digital twin technology is also gaining traction in the realm of healthcare infrastructure and operational management. Beyond patient-specific applications, digital twins are being used to optimize hospital workflows, resource allocation, and facility management. For example, digital twins can simulate hospital environments to predict patient flow, manage staffing needs, and optimize the use of medical equipment [12]. Furthermore, the integration of digital twins with multi-agent systems offers promising approaches to managing complex healthcare scenarios, such as severe trauma cases, by providing real-time decision support and coordination among various healthcare providers [13]. Additionally, digital twins are being explored for their potential in public health, where they can simulate the spread of infectious diseases, enabling health authorities to plan and implement effective containment strategies [14]. These applications demonstrate the versatility of digital twin technology in enhancing not only patient care but also the overall efficiency and responsiveness of healthcare systems.

Bibliometric analysis is defined as a computer-aided, quantitative assessment of publications in a particular field of scientific research [15]. Statistical analysis detects the core authors and research, as well as the links between them, and provides information on an author's productivity and influence [16,17]. It evaluates the impact factors of journals by describing the links among these publications and assists in strategic planning and funding decisions. Information is gathered from databases such as Web of Science, Scopus, or Google Scholar, cleaned to remove duplicates, and carefully analyzed [18]. Co-citation and co-authorship methods can show clusters of research and collaboration. Bibliometric techniques are essential for researchers, policymakers, and librarians, and support good navigation in the scientific literature [19].

RStudio is an integrated environment for the R programming language used for statistical computing and graphics. It has a desktop version, RStudio Desktop, and another one, called RStudio Server, running on a remote server accessed by the web browser [20]. Bibliometrix is a free open-source application written in R that is able to perform a visual representation of the scientific literature. It is a user-friendly tool that supports importing bibliographic information from several databases, running bibliometric analysis, and constructing data matrices for many study types [21,22]. Several types of visualization options are possible; therefore, it has become essential not only for scholars but also for librarians.

VOSviewer is a software tool developed for visualizing and analyzing bibliometric data. It can generate the co-authorship, co-citation, and keyword co-occurrence network and also visualize it. Offering both a user-friendly interface and an option for custom-made visualizations, VOSviewer aims to assist users in uncovering complex relationships within bibliographic databases in search of hidden patterns and trends [23,24]. Its rich functionality and user-oriented design turn it into an essential tool for conducting bibliometric analysis across a wide range of modern-day disciplines.

The research serves to map the research landscape of a digital twin in healthcare, together with an overview of applications, advancement, and trends in research identified. In this paper, an attempt will be made to decipher significant authors, leading sources, and significant research themes in this discipline through bibliometric analysis. Besides that, the study showcases the developmental nature of digital twin research in healthcare by pinpointing milestone identification, salient collaboration patterns, and recently emerging topics of interest. This would, in the longer term, provide viable insights for scholars, decision-makers, and practitioners in establishing a greater understanding of the contemporary status and prospects of digital twin technology in healthcare.

For this review, relevant scientific literature was systematically retrieved from the Scopus database up to June 29, 2024. Search terms "Digital Twin" AND "Health" OR "Medicine" were set up to retrieve documents published between 2012 and 2024; no language restriction was applied. Since the objective was to give a broad and deep analysis, several types of documents have been used, such as articles, conference papers, and book chapters. For that reason, only documents dealing explicitly with either discussing or evaluating digital twins in healthcare were included. The study excluded letters, reviews, and surveys to direct the research toward original studies. Likewise, any document lacking some essential bibliographic elements, such as author names or titles, was also excluded. The records retrieved in this research were managed in CSV files with full records and cited references. These were used for analysis with Biblioshiny and VOSviewer tools. Table 1 summarizes the results; it describes relevant papers, including duration of publication, sources used, average citations per document, total authors, and other pertinent metrics, that give a complete overview of the dimensions surveyed in the literature. “Document average age" refers to the average number of years since the publication of the documents included in the dataset, indicating the recency of the research. "Author's keywords (DE)" are the keywords provided by the authors to describe the content of their articles, while "Keywords plus (ID)" are additional relevant keywords generated from the titles of the cited references, offering an expanded view of the research topics covered.

**Table 1:** Overall information about the articles. Timespan: the period during which the documents were published (2012 to 2024). Sources (journals, books, etc.): the number of different publication sources, such as journals, books, and conference proceedings. Documents: the total number of documents analyzed in the study. Annual growth rate (%): the average annual growth rate of publications during the specified timespan. Document average age: the average age of the documents at the time of analysis. Average citations per document: the average number of citations received per document. References: the total number of references cited in all the documents. Keywords plus (ID): keywords assigned to articles by an indexing algorithm, providing additional terms related to the content. Author's keywords (DE): keywords specified by the authors of the documents. Authors: the total number of unique authors who have contributed to the documents. Authors of single-authored documents: the number of authors who have written documents alone. Single-authored documents: the number of documents written by a single author. Co-authors per document: the average number of co-authors per document. International co-authorships (%): the percentage of documents that have authors from multiple countries. Articles: the number of articles published in journals. Book chapters: the number of book chapters included in the analysis. Conference papers: the number of conference papers included in the analysis.

Description	Results
Main information about data	
Timespan	2012 to 2024
Sources (journals, books, etc.)	900
Documents	1663
Annual growth rate %	44.15
Document average age	1.73
Average citations per document	12.36
References	57,180
Document contents	
Keywords plus (ID)	10,369
Author's keywords (DE)	4,265
Authors	
Authors	5,592
Authors of single-authored documents	86
Authors collaboration	
Single-authored docs	95
Co-authors per document	4.5
International co-authorships %	25.62
Document types	
Article	844
Book chapter	125
Conference paper	694

## Review

Annual scientific production

Table 2 highlights the annual scientific production in the field of digital twins in healthcare from 2012 to 2021. The data reveal a gradual increase in publications over the years, with a notable surge beginning in 2018. Initial years show minimal activity, with only four articles in 2012 and none in 2013 and 2014. A slight increase occurred in 2015 and 2016 with two articles each, followed by a modest rise to six articles in 2017. The field gained significant momentum in 2018 with 14 articles, and a substantial jump to 62 articles in 2019. The trend continued with a dramatic increase to 115 articles in 2020 and 191 articles in 2021. This sharp rise in publications indicates growing interest and research activity in the application of digital twin technology within healthcare, reflecting its emerging importance and potential impact on the field.

**Table 2:** The annual scientific production from 2012 to 2024.

Year	No. of Articles
2012	4
2013	0
2014	0
2015	2
2016	2
2017	6
2018	14
2019	62
2020	115
2021	191

Most relevant sources

Table 3 highlights the most relevant sources for digital twin research in healthcare from 2012 to 2024. IEEE Access leads with 34 articles, followed by Sensors with 29 articles. Lecture Notes in Electrical Engineering and Lecture Notes in Networks and Systems each contributed 20 articles. Lecture Notes in Computer Science, including subseries on Artificial Intelligence and Bioinformatics, provided 19 articles. Lecture Notes in Civil Engineering added 17 articles, while Mechanical Systems and Signal Processing contributed 16 articles. The Journal of Physics: Conference Series featured 15 articles, Applied Sciences (Switzerland) had 13 articles, and Scientific Reports included 12 articles. These sources collectively provide a comprehensive view of the latest advancements and research trends in the application of digital twins in healthcare.

**Table 3:** Most relevant sources.

Sources	No. of Articles
IEEE Access	34
Sensors	29
Lecture Notes in Electrical Engineering	20
Lecture Notes in Networks and Systems	20
Lecture Notes in Computer Science (Including Subseries Lecture Notes in Artificial Intelligence and Lecture Notes in Bioinformatics)	19
Lecture Notes in Civil Engineering	17
Mechanical Systems and Signal Processing	16
Journal of Physics: Conference Series	15
Applied Sciences (Switzerland)	13
Scientific Reports	12

Most relevant authors

Table 4 presents the top 10 authors ranked by the number of articles published from 2012 to 2024 in the field of digital twins in healthcare. Liu Y and Zhang Y lead the list, each contributing 18 articles. Following closely, Liu Z and Wang Z each have 17 articles. Li Y and Wang Y have both authored 16 articles. Chen Y, Li J, and Li Z each contributed 15 articles, while Wang J has 14 articles. This distribution highlights the significant contributions of these authors to the evolving field of digital twins in healthcare, demonstrating their active involvement and influence in this area of research.

**Table 4:** Top 10 authors ranked by number of articles

Authors	No. of Articles
Liu Y	18
Zhang Y	18
Liu Z	17
Wang Z	17
Li Y	16
Wang Y	16
Chen Y	15
Li J	15
Li Z	15
Wang J	14

Trend topics

**Figure 1:**
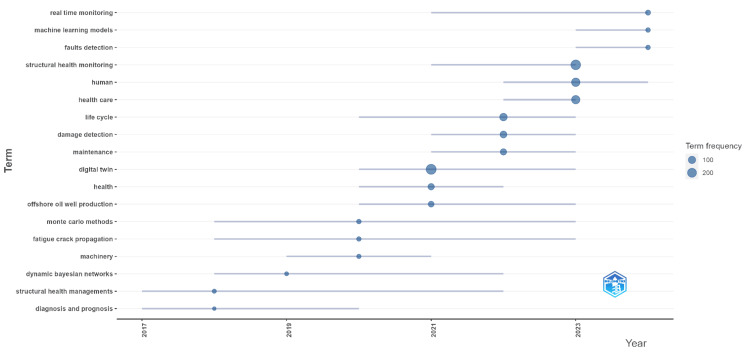
Trend topics in digital twins in healthcare Term frequency representation: the bubbles in the figure represent the frequency of each term within the specified time frame. The size of each bubble is proportional to the term's frequency. Frequency range: small bubbles indicate lower frequencies, starting from as few as one mention. Medium bubbles include terms with around 100 mentions. Large bubbles represent higher frequencies, such as 200 mentions. Maximum frequency: the largest bubble in the figure represents the highest frequency observed, with "digital twins" mentioned 288 times. Legend values: the legend provides reference points at 100 and 200 mentions for better understanding, but the size of each bubble corresponds to the frequency of term mentions, ranging from 1 to 288.

Figure 1 illustrates the trend topics in digital twins in healthcare from 2012 to 2024. The data show a significant rise in interest over the years, with terms such as "real-time monitoring," "machine learning models," and "fault detection" becoming increasingly prominent since 2017. "Structural health monitoring," "human," and "healthcare" terms started gaining frequency around 2018, reflecting growing applications in these areas. Notably, "digital twins" (288 mentions) and "life cycle" (111 mentions) began to gain traction around 2020, highlighting their emerging relevance. The trend also shows a notable increase in topics related to "health" (58 mentions) and "maintenance" (54 mentions) from 2020 onwards, indicating a broadening scope of research. More recently, topics such as "human" (166 mentions) and "healthcare" (160 mentions) have seen increased attention starting in 2022, emphasizing their growing importance in the field. The term frequencies are represented by varying bubble sizes, where larger bubbles indicate higher frequencies. While the legend provides examples of term frequencies at 100 and 200, the actual frequencies are not limited to these values. Instead, they represent a range of frequencies as observed in the data. This demonstrates that some topics have become central to the field, signifying their importance in ongoing research and development.

Three-field plot

**Figure 2:**
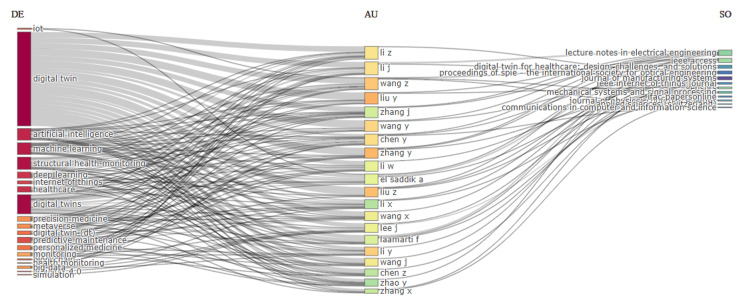
Three field Plot visualizing the relationship between keywords (DE), authors (AU), and sources (SO)

Figure 2 presents a three-field plot visualizing the relationship between keywords (DE), authors (AU), and sources (SO) in the context of digital twins in healthcare from 2012 to 2024. The plot reveals that "digital twin," "artificial intelligence," and "machine learning" are the most frequently occurring keywords, indicating their central role in this research area. Key authors such as Li Z, Liu Y, and Wang Z are prominently linked to these keywords, showcasing their significant contributions to the field. The authors are associated with various sources, including "Lecture Notes in Electrical Engineering," "Digital Twin for Healthcare: Design, Development, and Applications," and "Proceedings of SPIE - The International Society for Optical Engineering." This visualization highlights the interdisciplinary nature of research on digital twins in healthcare, with connections spanning across diverse topics, authors, and publication venues. The strong interconnections suggest a collaborative research environment, where influential authors contribute to multiple key topics and disseminate their findings through a variety of academic journals and conference proceedings.

Co-occurrence of keywords

**Figure 3:**
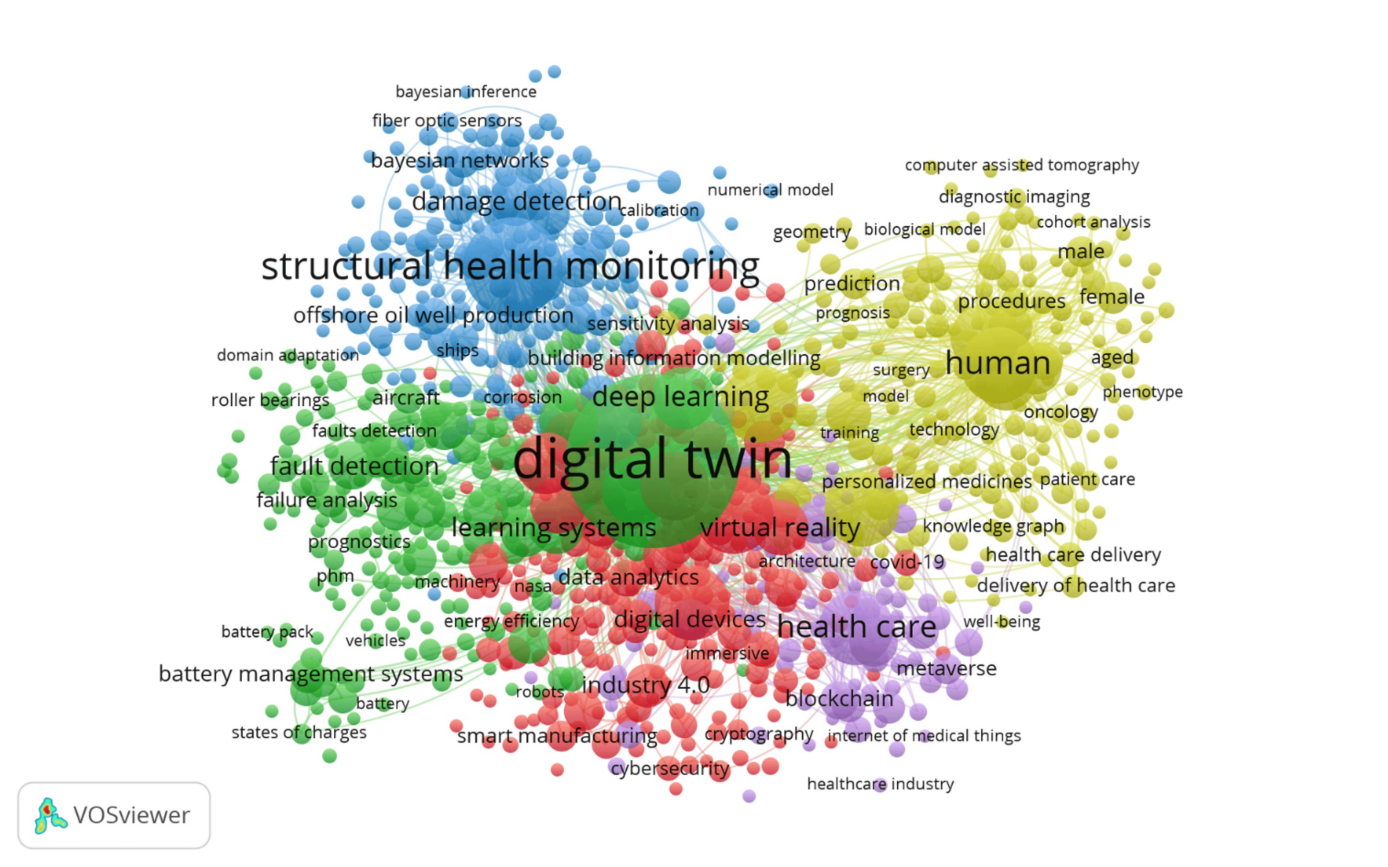
Co-occurrence of keywords Nodes: each node represents keywords related to digital twin research in healthcare; size indicates the frequency of the keyword's occurrence in the literature, with larger nodes representing more frequently occurring keywords. Colors: the blue cluster includes keywords related to "structural health monitoring" and "damage detection," the red cluster centers around "deep learning," "machine learning," and "data analytics," the green cluster focuses on "healthcare," "virtual reality," and "smart manufacturing," and the yellow cluster highlights keywords such as "human," "healthcare delivery," and "personalized medicine." Connecting lines: each line represents the co-occurrence relationships between keywords, with thicker lines indicating stronger relationships, meaning the connected keywords appear together more frequently in the literature.

Figure 3 presents a co-occurrence network map that visualizes the relationships between keywords in digital twin research in healthcare from 2012 to 2024, generated using the VOSviewer software. In this map, each node represents a keyword, with the size of the node indicating the frequency of the keyword's occurrence in the literature, with larger nodes denoting more frequently occurring keywords. The map is divided into clusters, each represented by a different color, grouping keywords that are often mentioned together. The blue cluster includes keywords related to "structural health monitoring" and "damage detection," indicating their importance in health system maintenance. The red cluster centers around "deep learning," "machine learning," and "data analytics," emphasizing the integration of advanced computational techniques in digital twin applications. The green cluster focuses on "healthcare," "virtual reality," and "smart manufacturing," illustrating the diverse application areas of digital twin technology. The yellow cluster, with keywords such as "human," "healthcare delivery," and "personalized medicine," highlights the growing interest in patient-centered applications and the enhancement of healthcare services through digital twins. The connecting lines between nodes represent co-occurrence relationships, with thicker lines indicating stronger relationships, meaning the connected keywords appear together more frequently in the literature. This visualization underscores the multidisciplinary nature of digital twin research in healthcare, reflecting the convergence of engineering, computer science, and medical fields to advance healthcare solutions, with "digital twin" prominently connected to a wide range of terms, highlighting its integral role in the research landscape.

Co-authorship between countries

**Figure 4:**
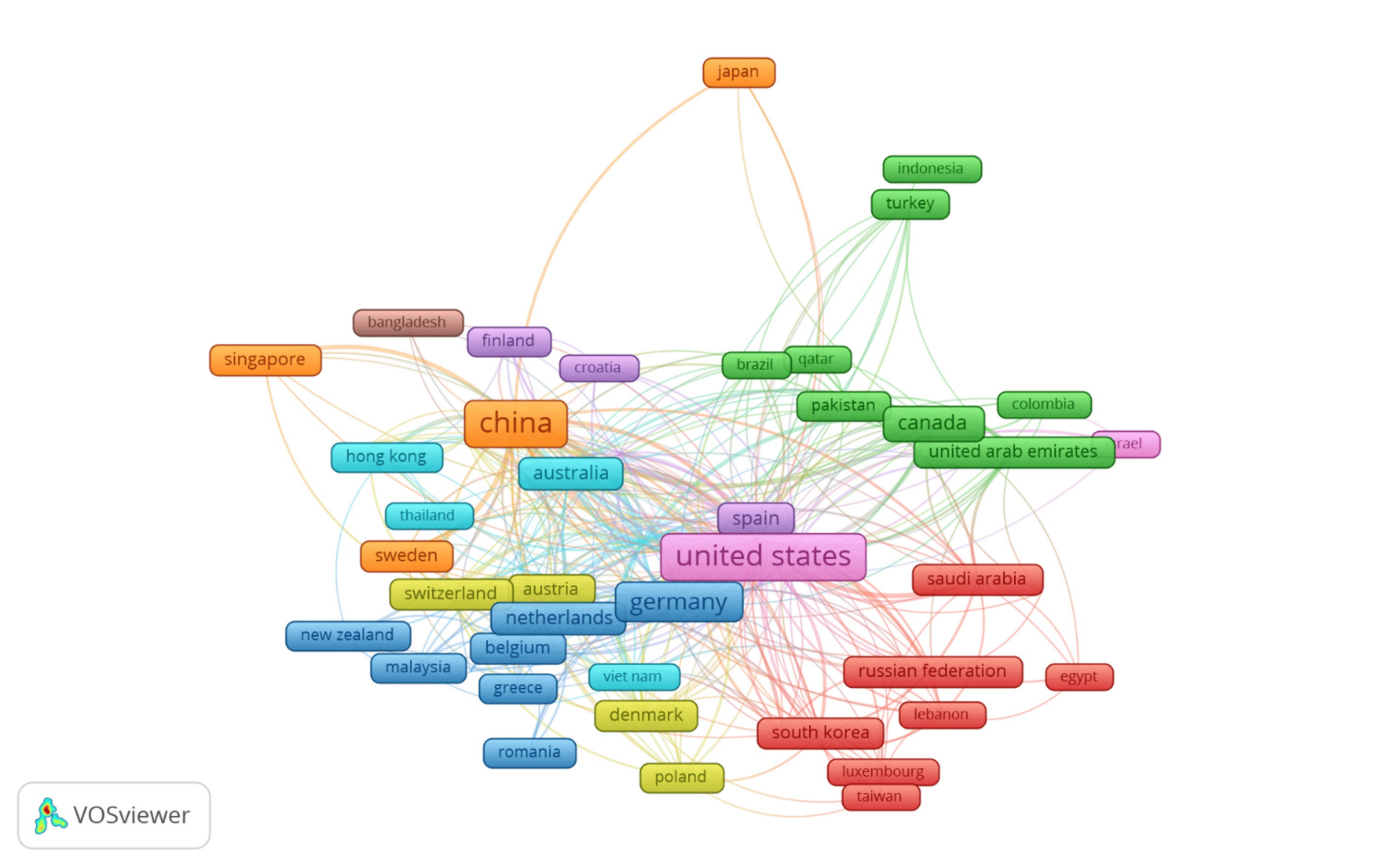
Co-authorship of countries Nodes/boxes: each node or box represents a country involved in digital twin research in healthcare. The size of the nodes/boxes indicates the number of publications or the level of activity in digital twin research for each country. Larger nodes signify more publications or higher activity. Colors: different colors represent clusters or groups of countries that frequently collaborate with each other. For example, nodes with the same color belong to the same cluster and indicate strong collaborative ties within that group. Connecting lines: the lines between nodes represent co-authorship links, indicating collaboration between researchers from different countries. Thicker lines indicate stronger or more frequent collaboration, while thinner lines suggest weaker or less frequent collaboration.

Figure 4 illustrates the co-authorship network of countries in digital twin research in healthcare from 2012 to 2024. Different colors are used to group countries into clusters based on their collaborative relationships. Each color represents a cluster of countries that frequently collaborate with each other. The size of a node is proportional to the country's involvement in digital twin research in healthcare. Larger nodes signify countries with greater contributions to the field. The lines connecting the nodes represent co-authorship links between countries. A line between two nodes indicates that researchers from these countries have collaborated on one or more research papers. The thickness of the lines signifies the strength of the collaboration, with thicker lines indicating a higher number of co-authored papers and stronger collaborative relationships. The United States, China, and Germany are prominent nodes, indicating their leading roles in this field. Strong collaborative links are observed between these countries, as well as with other major contributors such as Canada, the United Kingdom, and Australia. The network shows a high level of international collaboration, with numerous interconnections between countries across different continents. For instance, European countries such as the Netherlands, Sweden, and Spain have multiple collaborative ties with both the United States and China. Similarly, Asian countries such as Japan, South Korea, and Singapore also show strong links with these leading nations. This visualization highlights the global nature of digital twin research in healthcare, with significant contributions and partnerships spanning North America, Europe, and Asia. The dense web of connections suggests a robust international research community working collaboratively to advance the field, leveraging diverse expertise and resources from around the world.

Research gaps and practical implications

Although digital twins in health are very promising, several research gaps exist. Among these is the challenge of interoperability between heterogeneous data sources. Digital twin systems will require the integration of real-time data streaming from wearable devices, EHRs, imaging modalities, and genomic profiling. This places an additional burden on standardized protocols and robust governance frameworks for data. Hence, if data are not integrated seamlessly, this diminishes the potential uses of digital twins and thus affects their clinical effectiveness.

The first and foremost are concerns about data privacy and security. Since digital twins continuously collect data to analyze it, they have essential elements such as patient confidentiality protection and measures concerning cyber threat security. This has to be attended to by future research, with advanced encryption methodologies and the development of highly secure data management systems. Another research gap, in particular, has to do with the validation and generalization of digital twin models. Provided that the bulk of studies demonstrate potential for any given application of a digital twin in one scenario or another, there is a definite requirement for large-scale, multi-center trials that confirm their effectiveness across different populations of patients and dissimilar clinical conditions. That would set up the generalizability of such models and reveal their implicit limitations and biases, which are inborn in their very design.

From a practical point of view, implementing digital twin technology in healthcare settings is such a vast and expensive logistic task. High-performance computing systems and highly evolved sensors are just two examples of infrastructural acquisitions required for a healthcare provider to digest and analyze real-time data. Finally, significant training of healthcare providers is necessary if they are ever to feel comfortable using such technologies in situating digital twins within present clinical workflows.

In addition to the aforementioned challenges, this study is limited by its focus on the Scopus database, which may not encompass the entirety of relevant literature on digital twins in healthcare. The bibliometric analysis, being inherently more quantitative, might overlook nuanced insights found in qualitative studies or those indexed in other databases. Future research should aim to incorporate a broader range of sources to provide a more comprehensive understanding of the limitations and potential of digital twins in healthcare.

## Conclusions

The rise of digital twins in healthcare shows a sea change in medical practice and opens new avenues for personalized medicine, real-time monitoring, and predictive analytics. This bibliometric analysis underlines the fast growth and scope of research within this domain and highlights interdisciplinary collaboration driving innovation in digital twin technology. Though development is promising in this area, it is still haunted by many challenges and research gaps that need to be overcome to apply digital twin technology effectively. Interoperability of data, enhancement of privacy and security of data, model validation across divergent populations, and practical implementation issues are some critical lines of inquiry for future research and development. Digital twin technology is one such constituent that is expected to play a central role in healthcare shortly by most efficiently improving diagnosis accuracy, optimizing treatment plans, and consequently changing patient outcomes for good. It is only when these challenges are met and the opportunities of this technology are availed that a shift of strategies by healthcare providers toward proactive and personalized care can be achieved.
